# Preoperative complications in children with mesenteric lymphatic malformations: Incidence, risk factors and outcomes

**DOI:** 10.3389/fped.2022.1033897

**Published:** 2022-09-29

**Authors:** Jiayu Yan, Li Wang, Chuanping Xie, Chunhui Peng, Wenbo Pang, Yajun Chen

**Affiliations:** ^1^ Department of General Surgery, Beijing Children’s Hospital, Capital Medical University, National Center for Children’s Health, Beijing, China; ^2^ Department of Emergency Surgery, Beijing Children’s Hospital, Capital Medical University, National Center for Children’s Health, Beijing, China

**Keywords:** mesenteric lymphatic malformations, children, preoperative complication, hemorrhage, infection, intestinal volvulus, risk factor

## Abstract

**Background:**

Preoperative complications of mesenteric lymphatic malformations (ML) in children are various and complex. We aim to analyze the incidences and risk factors of three major preoperative complications (hemorrhage of the cyst, infection of the cyst and intestinal volvulus) in ML patients, and explore their influence on the outcomes.

**Methods:**

This retrospective cohort study enrolled ML patients undergoing surgery at Beijing Children's Hospital between June 2016 and June 2022 and classified them according to different preoperative complications, preoperative hemorrhage or infection, and preoperative intestinal volvulus. The groups were examined and compared according to sex, age at admission, presenting symptoms, laboratory examinations, imaging examinations, preoperative treatments, cyst characteristics, surgical details, perioperative clinical data, and follow-up. Logistic regression analysis was performed to identify the independent risk factors for preoperative hemorrhage or infection, and preoperative intestinal volvulus.

**Results:**

Of the 104 enrolled ML patients, 27 (26.0%) had preoperative hemorrhage or infection, and 22 (21.2%) had preoperative intestinal volvulus. Univariate analysis showed that patients with preoperative hemorrhage or infection had a higher rate of ML in the mesocolon (44.4 vs. 23.4%, *p* < 0.038) and larger cysts (10 vs. 8 cm, *p* = 0.042) than patients without preoperative hemorrhage or infection. Multivariable logistic regression analysis found that the location (OR, 3.1; 95% CI, 1.1–8.6; *p* = 0.026) and size of the cyst (≥7.5 cm) (OR, 6.2; 95% CI, 1.6–23.4; *p* = 0.007) were independent risk factors for preoperative hemorrhage or infection. Preoperative intestinal volvulus was only found in ML at the intestinal mesentery. Further analysis showed that ML in the jejunal mesentery was an independent risk factor for preoperative intestinal volvulus (OR, 3.3; 95% CI, 1.1–10.0; *p* = 0.027). Patients with preoperative hemorrhage or infection spent more on hospitalization costs than patients without preoperative hemorrhage or infection (3,000 vs. 2,674 dollars, *p* = 0.038).

**Conclusions:**

ML patients should be treated as soon as possible after diagnosis. The location and size of the cyst were independent risk factors for preoperative hemorrhage or infection. ML in the jejunal mesentery was an independent risk factor for preoperative intestinal volvulus.

## Introduction

Mesenteric lymphatic malformations (ML) are rare lesions and account for nearly 1%–5% of all lymphatic malformations (1, 2). ML are benign vascular lesions almost associated with abnormal embryonic development of the lymphatics but may partly be explained by the bleeding or inflammation in the lymphatic channels (3, 4). The reported incidence of ML was approximately 1/250,000–1/20,000, with diagnosis around 60% before the fifth year of life (4, 5). Most ML patients are initially asymptomatic or have vague abdominal symptoms, which can be aggravated when the cysts increase in size or develop complications. Moreover, children with ML are more prone to associated complications and present with acute symptoms (4).

The preoperative complications of ML are various and complex, including hemorrhage, infection and rupture of the cyst, intestinal volvulus, and some rare complications, such as intussusception and incarcerated hernia, which can affect the choice of treatment and result in a poor prognosis (6–11). Previous reports have shown some preoperative complications of ML, but they are sparse. No study has described the incidences and risk factors of preoperative complications of ML and explored their influence on the outcomes of patients.

Herein, we focused on three major preoperative complications of ML (hemorrhage of cyst, infection of the cyst and intestinal volvulus) and conducted a 6-year single-center retrospective study to analyze the risk factors and influence of the above preoperative complications in children with ML.

## Materials and methods

### Patient selection and clinical data

After approval by the Ethics Committee of Beijing Children's Hospital (approval number [2022]-E-131-R), the medical records of children diagnosed with mesenteric lymphangioma or mesenteric cysts and admitted to Beijing Children's Hospital, Children's National Medical Center, China, between June 2016 and June 2022 were retrospectively reviewed. Informed consent was waived due to the nature of the study. Patients who underwent surgery at our center and were confirmed with ML by postoperative histopathology were included. Patients who underwent surgery at other hospitals before our admission, those with only radiologic evidence of ML, and those confirmed with combined vascular malformations, were excluded.

From June 2016, the medical files of all patients can be obtained from the medical record system. We retrospectively collected clinical data of ML patients from the inpatient and outpatient medical files. The clinical data included sex, age at admission, presenting symptoms, preoperative complications, laboratory examinations, imaging examinations, preoperative treatments, surgical details, pathological data, and perioperative clinical data. Telephone interviews were performed in August 2022 to ask the patients' parents about the child's postoperative complications.

### Study design

For the purpose of the present study, we focused on major preoperative complications of ML, including hemorrhage of cyst, infection of the cyst and intestinal volvulus, which were confirmed by reviewing the surgical records. According to the surgical records, the content of the cyst could contain serous, chylous, serosanguinous, or turbid fluid. Serosanguinous fluid represents cyst hemorrhage, and turbid fluid indicates cyst infection (4). However, hemorrhage and infection of cyst in ML patients are often concomitant and challenging to distinguish.

Through reviewing the surgical records, we also identified other cyst characteristics of ML, including the specific location, classification, pathological type, and size. The specific locations of ML varied depending on the mesentery involved. The ML classifications were based on a previous classification modified by Kim et al. in 2016 (1). The pathological types referred to the types described in previous studies, including macrocystic-type (≥1 cm), microcystic-type (<1 cm), and mixed cystic-type (12, 13). The size of the ML was defined as the length of the cyst.

For analysis, we divided the included ML patients into two groups based on the content of the cyst. The ML patients with preoperative hemorrhage or infection had serosanguinous fluid or turbid fluid of the cyst. In contrast, the patient did not have preoperative hemorrhage or infection. The risk factors of preoperative hemorrhage or infection were analyzed by clinical features, such as sex, age at admission, and cyst characteristics (specific locations, classification, pathological types, and size).

In addition, we also analyzed the risk factors of preoperative intestinal volvulus and evaluated the effects of the above three preoperative complications on treatments and outcomes of ML patients. The evaluation of treatments included whether preoperative conservative treatment or emergency surgery was performed, which surgical approach was chosen, and how long the operation lasted. The evaluation of outcomes included oral intake time, hospital stay, and postoperative complications. Hospitalization costs were also compared to understand the impact of preoperative complications on the financial burden of the patients' families.

### Statistical analysis

Continuous variables with a normal distribution are presented as the means ± standard deviations and were analyzed with Student's *t*-test. Continuous variables with a nonnormal distribution are presented as the median (interquartile range) and were analyzed with the Mann–Whitney test. Categorical variables are expressed as numbers (percentages) and were analyzed with the *χ*^2^ test or Fisher’s exact test. The receive operating characteristic curve (ROC curve) was used to determine the cut-off values. Multivariate analysis was performed with binary logistic regression. *P* < 0.05 (2-sided) was considered statistically significant. Statistical analysis was performed using IBM SPSS version 26 (SPSS Inc, Chicago, IL).

## Results

### Clinical features of ML in children

**Figure 1:**
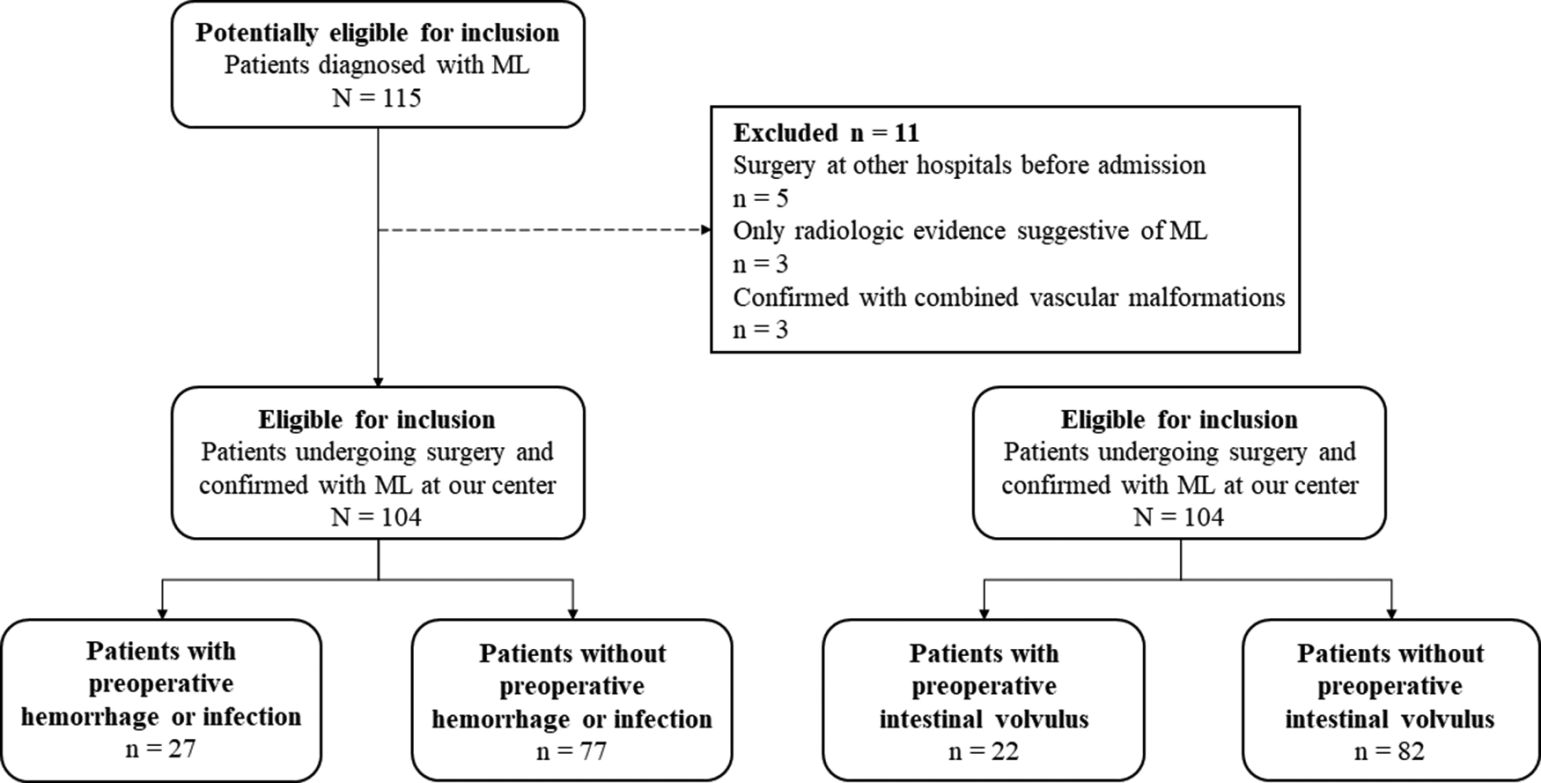
The flowchart of the inclusion, exclusion and group criteria. ML, mesenteric lymphatic malformations.

From June 2016 to June 2022, 115 patients diagnosed with ML were at our center for treatment. In total, 104 patients were enrolled in this study. As shown in Figure 1, according to different preoperative complications, the patients were divided into two groups: patients with preoperative hemorrhage or infection (27/104, 26.0%) and patients without preoperative hemorrhage or infection (77/104, 74.0%), patients with preoperative intestinal volvulus (22/104, 21.2%) and patients without preoperative intestinal volvulus (82/104, 78.8%). All included patients underwent the initial surgical treatment at our center.

As shown in Table 1, patients' median age at admission was 4.0 (2.3, 6.2) years, with 65.4% male and 34.6% female. The main symptoms were abdominal pain (76/104, 73.1%) and vomiting (51/104, 51.0%). Seventeen patients (17/104, 16.3%) received conservative observation after diagnosis of ML, then developed aggravated symptoms and underwent surgical treatment. Laboratory examinations before admission indicated infection or anemia in 34 patients (34/104, 32.7%). The preoperative imaging examinations mainly included abdominal ultrasound (104/104, 100.0%) and CT (69/104, 66.3%).

**Table 1:** Characteristics between ML patients with and without preoperative hemorrhage or infection.

Characteristics	Total	Hemorrhage or infection		*p*-value
	(*N* = 104)	Yes (*n* = 27)	No (*n* = 77)	
Sex				
Male	68 (65.4)	14 (51.9)	54 (70.1)	0.086
Female	36 (34.6)	13 (48.1)	23 (29.9)	
Age (years)	4.0 (2.3, 6.2)	3.8 (1.3, 7.7)	4.0 (2.4, 5.9)	0.801
<2	22 (21.2)	8 (29.6)	14 (18.2)	
2–6	53 (51.0)	9 (33.3)	44 (57.1)	0.102
≥7	29 (27.8)	10 (37.0)	19 (24.7)	
Symptoms				
Incidental diagnosis	5 (4.8)	0 (0.0)	5 (6.5)	0.323
Abdominal pain	76 (73.1)	21 (77.8)	55 (71.4)	0.522
Vomiting	53 (51.0)	12 (44.4)	41 (53.2)	0.431
Abdominal distention	40 (38.5)	18 (66.7)	22 (30.6)	0.001
Abdominal mass	25 (24.0)	11 (40.7)	14 (18.2)	0.018
Fever	25 (24.0)	12 (44.4)	13 (16.9)	0.004
Diarrhea	10 (9.6)	6 (22.2)	4 (5.2)	0.01
Preoperative observation				
Yes	17 (16.3)	8 (29.6)	9 (11.7)	0.030
No	87 (83.7)	19 (70.4)	68 (88.3)	
Laboratory examination^a^				
Normal	70 (67.3)	10 (37.0)	60 (77.9)	<0.001
Infection or anemia	34 (32.7)	17 (63.0)	17 (22.1)	
Imaging examinations				
Ultrasound	104 (100.0)	27 (100.0)	77 (100.0)	>0.999
CT	69 (66.3)	19 (70.4)	50 (64.9)	0.607
MRI	5 (4.8)	1 (3.7)	4 (5.2)	>0.999
Preoperative intestinal volvulus				
Yes	22 (21.2)	6 (22.2)	16 (20.8)	0.874
No	82 (78.8)	21 (77.8)	61 (79.2)	
Locations				
Small intestine	74 (71.2)	15 (55.6)	59 (76.6)	0.038
Colon	30 (28.8)	12 (44.4)	18 (23.4)	
Cyst size (cm)	10 (6, 14)	10 (8, 15)	8 (6, 13)	0.042
<7.5	33 (33.3)	3 (11.1)	30 (41.7)	0.004
≥7.5	66 (66.7)	24 (88.9)	42 (58.3)	
Classifications				
I	60 (57.7)	15 (55.6)	45 (58.4)	0.918
II	19 (18.3)	7 (25.9)	12 (15.6)	
III	14 (13.5)	3 (11.1)	11 (14.3)	
IV	11 (10.5)	2 (7.4)	9 (11.7)	
Pathological types				
Macrocystic-type	90 (86.5)	23 (85.2)	67 (87.0)	0.804
Microcystic-type	4 (3.9)	1 (3.7)	3 (3.9)	
Mixed cystic-type	10 (9.6)	3 (11.1)	7 (9.1)	

ML, Mesenteric lymphatic malformations.

^a^ The laboratory data collected included C-reactive protein, white blood cells, and hemoglobin. The patients with C-reactive protein >8 mg/L or white blood cells >10 × 10^9^/L were considered infected. The patients with hemoglobin <120 g/L were considered to be anemia.

### Risk factors for preoperative hemorrhage or infection

The sex and age at admission did not differ significantly between patients with and without preoperative hemorrhage or infection (Table 1). Patients with preoperative hemorrhage or infection had a higher rate of abdominal distention (66.7 vs. 30.6%, *p* = 0.001), abdominal mass (40.7 vs. 18.2%, *p* = 0.018), fever (44.4 vs. 16.9%, *p* = 0.004), and diarrhea (22.2 vs. 5.2%, *p* = 0.010). Moreover, 63.0% of patients with preoperative hemorrhage or infection had abnormal laboratory results (63.0 vs. 22.1%, *p* < 0.001).

**Figure 2:**
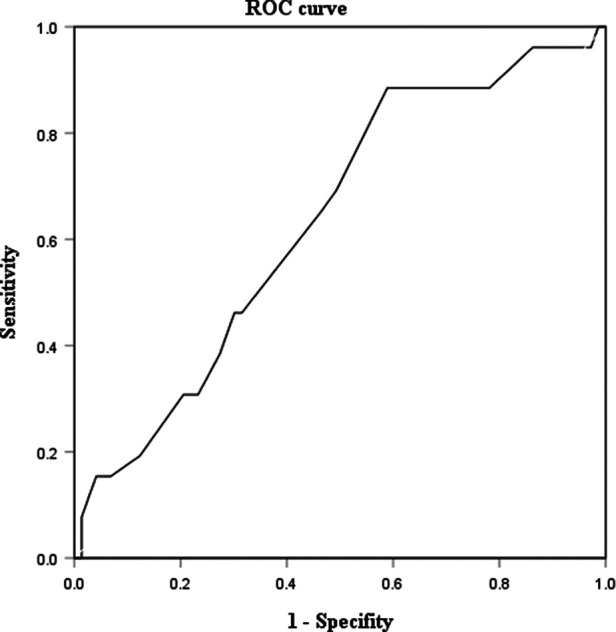
ROC curve of cyst size in ML patients. ROC curve, receive operating characteristic curve; ML, mesenteric lymphatic malformations.

The univariate analysis showed that patients with preoperative hemorrhage or infection had a higher rate of ML in the mesocolon (44.4 vs. 23.4%, *p* < 0.038) and larger cysts (10 vs. 8 cm, *p* = 0.042) than patients without preoperative hemorrhage or infection. The ROC curve of cyst size was analyzed. *P*-value of cyst size revealed statistical significance (*p* = 0.043). The AUROC of cyst size was 0.634 (Figure 2). Using the Jordan index, the optimal cut-off value of cyst size was 7.5 cm, with a sensitivity of 0.885 and a specificity of 0.411. Further multivariable logistic regression analysis found that the location (OR, 3.1; 95% CI, 1.1–8.6; *p* = 0.026) and size of the cyst (≥7.5 cm) (OR, 6.2; 95% CI, 1.6–23.4; *p* = 0.007) were independent risk factors for preoperative hemorrhage or infection (Table 2).

**Table 2:** Multivariable logistic regression analysis of risk factors for preoperative hemorrhage or infection in ML patients.

Risk factors	OR (95% CI)	*p*-value
Locations	3.1 (1.1–8.6)	0.026
Cyst size	6.2 (1.6–23.4)	0.007

### Risk factors for preoperative intestinal volvulus

According to the surgical findings of intestinal volvulus, the included patients were divided into two groups: patients with or without preoperative intestinal volvulus. As shown in Table 3, compared with the patients without preoperative intestinal volvulus, the patients with preoperative intestinal volvulus had a higher rate of abdominal pain (90.9 vs. 68.3%, *p* = 0.034) and vomiting (86.4 vs. 41.5%, *p* < 0.001), and a lower rate of fever (0.0 vs. 30.5%, *p* = 0.001). Preoperative intestinal volvulus was only found in patients with ML at the intestinal mesentery. Further analysis showed that an ML in the jejunal mesentery was an independent risk factor for preoperative intestinal volvulus (OR, 3.3; 95% CI, 1.1–10.0; *p* = 0.027) Table 4).

**Table 3:** Characteristics between ML patients with and without preoperative intestinal volvulus.

Characteristics	Intestinal volvulus		*p*-value
	Yes (*n* = 22)	No (*n* = 82)	
Sex			
Male	15 (51.9)	53 (70.1)	0.086
Female	7 (48.1)	29 (29.9)	
Age (years)	4.5 (1.9, 7.8)	4.0 (2.0, 5.8)	0.34
<2	2 (9.1)	20 (24.4)	
2–6	11 (50.0)	42 (51.2)	0.059
≥7	9 (40.9)	20 (24.4)	
Symptoms			
Incidental diagnosis	0 (0.0)	5 (6.1)	0.582
Abdominal pain	20 (90.9)	56 (68.3)	0.034
Vomiting	19 (86.4)	34 (41.5)	<0.001
Abdominal distention	7 (31.8)	33 (40.2)	0.471
Abdominal mass	7 (31.8)	18 (22.0)	0.336
Fever	0 (0.0)	25 (30.5)	0.001
Diarrhea	0 (0.0)	10 (12.2)	0.115
Cyst size (cm)	12 (8, 15)	9 (6, 13)	0.086
Locations			
Small intestine	22 (100.0)	52 (63.4)	<0.001
Colon	0 (0.0)	30 (36.6)	
Classifications			
I	16 (72.8)	44 (53.7)	0.327
II	0 (0.0)	19 (23.2)	
III	3 (13.6)	11 (13.4)	
IV	3 (13.6)	8 (9.7)	
Pathological types			
Macrocystic-type	19 (86.4)	71 (86.6)	0.915
Microcystic-type	0 (3.7)	4 (4.9)	
Mixed cystic-type	3 (13.6)	7 (8.5)	

ML, Mesenteric lymphatic malformations.

**Table 4:** Univariate analysis of risk factor for preoperative intestinal volvulus in ML patients.

Risk factor	Intestinal volvulus		OR (95% CI)	*p*-value
	Yes (*n* = 22)	No (*n* = 52)		
Location	22	41 (11 unacquirable)	3.3 (1.1–10.0)	0.027
Jejunum	15 (68.2%)	16 (39.0%)		
Ileum	7 (31.8%)	25 (61.0%)		

### Treatments and outcomes of ML in children

As shown in Table 5, patients with preoperative hemorrhage or infection were more likely to receive conservative treatments after admission (37.0 vs. 7.8%, *p* < 0.001) than patients without preoperative hemorrhage or infection, and patients with preoperative intestinal volvulus were more likely to undergo emergency surgery than patients without preoperative intestinal volvulus (45.5 vs. 6.1%, *p* < 0.001). The preoperative complications did not influence the choice of surgical approach. However, the operation time in patients with preoperative intestinal volvulus was longer than in those without preoperative intestinal volvulus (139 vs. 105 min, *p* = 0.006).

**Table 5:** Comparison of treatments and outcomes in ML patients with or without preoperative complications.

	Hemorrhage or infection		*p*–value	Intestinal volvulus		*p*-value
	Yes (*n* = 27)	No (*n* = 77)		Yes (*n* = 22)	No (*n* = 82)	
Conservative treatment, *n* (%)						
Yes	10 (37.0)	6 (7.8)	<0.001	3 (13.6)	13 (15.9)	>0.999
No	17 (63.0)	71 (92.2)		19 (86.4)	69 (84.1)	
Emergency surgery, *n* (%)						
Yes	6 (22.2)	9 (11.7)	0.18	10 (45.5)	5 (6.1)	<0.001
No	21 (77.8)	68 (88.3)		12 (54.5)	77 (93.9)	
Surgical approach, *n* (%)						
Laparotomy^a^	17 (63.0)	41 (53.2)	0.382	15 (68.2)	43 (52.4)	0.187
Laparoscopy^b^	10 (37.0)	36 (46.8)		7 (31.8)	39 (47.6)	
Operation time, mins	130 (57, 165)	110 (80, 145)	0.187	139 (114, 166)	105 (75, 145)	0.006
Oral intake time, days	4 (2, 5)	3 (3, 4)	0.422	4 (4, 4)	4 (2, 4)	0.024
Total hospital stay, days	9 (8, 16)	9 (8, 11)	0.059	8 (8, 10)	9 (8, 12)	0.317
Postoperative hospital stay, days	7 (6, 8)	6 (5, 7)	0.056	7 (7, 7)	6 (5, 7)	0.038
Hospitalization costs, dollars	3,000 (2589, 3654)	2,674 (2133, 3161)	0.038	2,754 (2384, 3138)	2,682 (2143, 3253)	0.899
Postoperative complications, *n* (%)	2 (7.4)	11 (14.3)	0.505	1 (4.5)	12 (14.6)	0.291

ML, Mesenteric lymphatic malformations.

^a^ Including the cases underwent conversion from laparoscopy to open surgery.

^b^ Including one case undergoing robotic surgery.

**Figure 3:**
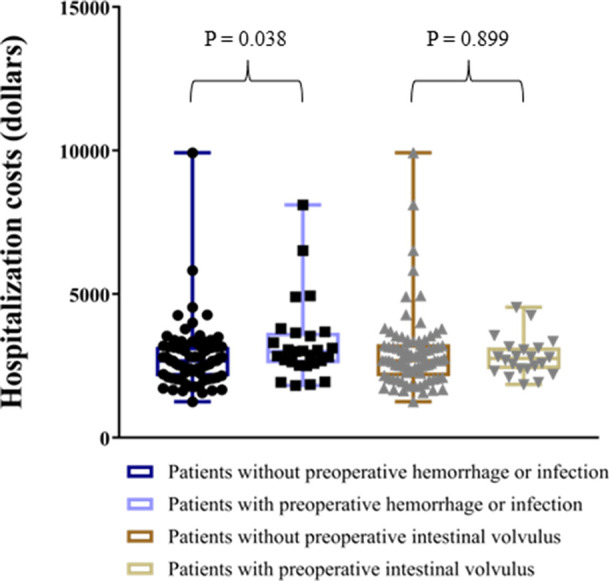
Hospitalization costs of ML patients. ML, mesenteric lymphatic malformations.

As for outcomes, the oral intake time and the length of hospital stay did not differ between patients with and without preoperative hemorrhage or infection. However, patients with preoperative intestinal volvulus needed a longer time to resume oral intake (*p* = 0.024) and had a longer hospital stay (*p* = 0.038) after surgery. Thirteen patients (13/104, 12.5%) had postoperative complications, including 6 with adhesive ileus, 5 with residual or recurrence of cyst, 1 with incisional hernia, and 1 with intussusception, which were not influenced by preoperative complications. In addition, patients with preoperative hemorrhage or infection spent more on hospitalization costs than patients without preoperative hemorrhage or infection (3000 vs. 2,674 dollars, *p* = 0.038) (Figure 3).

## Discussion

In the present study, we reported the incidences of three preoperative complications (hemorrhage of the cyst, infection of the cyst and intestinal volvulus) of ML in children, and explored the risk factors of the above preoperative complications in a cohort of 104 patients. Based on logistic regression analysis, we showed that the location and size of the cyst were independent risk factors for preoperative hemorrhage or infection. In addition, ML in the jejunal mesentery was an independent risk factor for preoperative intestinal volvulus.

A review of our data showed that the sex distribution and age at diagnosis of ML in children were similar to that reported in previous studies (1, 4, 14). The initial presenting symptoms of ML are often nonspecific, including obscure abdominal discomfort and mild abdominal pain, which could resolve spontaneously (4). Therefore, considering that ML were benign, some patients chose conservative observation after diagnosis in our study. However, ML tend to grow proportionately as the child grows and ultimately become symptomatic due to developing complications, which result in some apparent symptoms, such as vomiting, abdominal distention, and abdominal mass (15, 16). As observed in our study, patients with preoperative hemorrhage or infection had a higher rate of abdominal distention, abdominal mass, fever, and diarrhea, and correspondingly they had abnormal laboratory results. Severe hemorrhage may cause acute abdominal pain, even a bowel obstruction or signs of peritonitis due to space-occupying and compression effects of mass (6). In addition, almost all patients with preoperative volvulus in our study had abdominal pain (90.9%) and intermittent bilious vomiting (86.4%) (9). These results suggested that preoperative complications should be considered when severe or new symptoms occurred, and imaging examinations should be performed to determine the subsequent treatments. Several studies have found that ultrasound and CT are the most commonly used imaging examinations for the preoperative diagnosis of ML, which can also help to detect preoperative complications (1, 14, 17).

The preoperative complications of ML have been previously reported. Traubici J et al. retrospectively reviewed 2 ML patients with preoperative volvulus, and Sriram G et al. presented 1 colonic ML complicated by abscess formation (6, 7). However, the incidences of all preoperative complications of ML have not been reported. Our study reported that the incidences of preoperative hemorrhage or infection and preoperative intestinal volvulus in ML patients were 26.0% (27/104) and 21.2% (22/104), respectively. On the one hand, the above preoperative complications were relatively common in enrolled ML patients. On the other hand, patients who developed the above preoperative complications required emergency treatments (1, 4). For example, patients with preoperative hemorrhage or infection need hemostatic agents or antibiotics, and patients with volvulus are considered to undergo emergency surgery.

As observed in our study, compared with patients without preoperative hemorrhage or infection, patients with preoperative hemorrhage or infection had a higher rate of ML in the mesocolon (44.4%) but still predominantly in the intestinal mesentery. This may be related to the fact that ML are mostly located in the small intestine (14). Of more concern was a significantly larger length of the cysts in patients with preoperative hemorrhage or infection. This condition was mainly due to the acute fluid collection of the cysts in these patients. Hemorrhage or infection could stimulate the cyst wall to secrete fluid, further resulting in the enlargement of the cyst. Although the AUROC of cyst size in our study was 0.634, this would be high enough when combined with clinical symptoms and laboratory examinations in clinical practice (18). Therefore, when a cyst ≥7.5 cm in length is found in ML patients on preoperative examinations, the patients could be considered to develop hemorrhage or infection in combination with obvious symptoms and abnormal laboratory results. In these patients, conservative treatments, such as necessary transfusion, fluid rehydration and anti-infective therapy, should be performed before surgery to reduce surgical complications (7).

Interestingly, preoperative intestinal volvulus was only found in patients with ML at the intestinal mesentery. Further analysis revealed that preoperative intestinal volvulus occurred in half of ML in the jejunal mesentery (15/31, 48.4%). This finding might be related to the fact that the ML in the jejunal mesentery is close to the root of the mesentery, and some cysts affect the normal peristalsis of the intestine, resulting in a “twisted effect” similar to congenital intestinal malrotation (19). If intestinal volvulus was suspected in ML patients, the surgeons had to examine the abdomen, restore the existing volvulus, and further observe the bowel blood supply and peristaltic function. Thus, the operation time of patients with preoperative intestinal volvulus in our study was longer. In addition, we found that ML patients with intestinal volvulus did not seem to have severe intestinal ischemia and necrosis. For patients with suspected preoperative intestinal volvulus by imaging examinations, if the symptoms are mild, comprehensive preoperative evaluation should be performed to select the appropriate surgical approach and methods according to the site and size of ML (1, 14).

Complete surgical resection is the standard treatment of ML with a good prognosis (4). Some previous studies have shown that the specific surgical method for ML is chosen mainly depending on the classification of the cyst (1, 4). When patients have type IV ML or complex ML, surgery alone is unable to remove the lesion completely, and systemic medications and sclerotherapy are required before or after surgery. However, the long-term prognosis of these treatments is unknown (15). In our study, there were 5 ML patients with residual or recurrence of cyst after surgery. Although they have no obvious symptoms, a regular follow-up is still necessary to prevent complications and give them timely treatment.

The last point that we have observed is the differences in hospitalization costs between patients with and without preoperative hemorrhage or infection. It could be explained by the additional costs of medications during conservative treatments in patients with preoperative hemorrhage or infection. Although asymptomatic lymphatic malformations at other sites can be treated conservatively, no study has reported the efficacy of expectant treatment in ML patients (20). Therefore, to avoid related preoperative complications, we recommended that ML patients be treated as soon as possible after diagnosis rather than conservative observation, even if they are asymptomatic.

This study has several limitations. First, the retrospective design of the study could lead to some missing data. This is particularly true for some data that were not systematically recorded (history of previous infections, external force), limiting the possibility to analyze those items. Secondly, the study could not distinguish between patients with preoperative hemorrhage and patients with preoperative infection, which would extend the applicability of our findings. Finally, the study did not analyze the influence of preoperative complications on surgical methods because there were many interference factors, such as different surgeons and emergency surgeries.

## Conclusion

ML patients should be treated as soon as possible after diagnosis. The location and size of the cyst were independent risk factors for preoperative hemorrhage or infection. ML in the jejunal mesentery was an independent risk factor for preoperative intestinal volvulus.

## Data Availability

The original contributions presented in the study are included in the article/Supplementary Material, further inquiries can be directed to the corresponding author/s.
